# Zearalenone altered the cytoskeletal structure via ER stress- autophagy- oxidative stress pathway in mouse TM4 Sertoli cells

**DOI:** 10.1038/s41598-018-21567-8

**Published:** 2018-02-20

**Authors:** Wanglong Zheng, Bingjie Wang, Mengxue Si, Hui Zou, Ruilong Song, Jianhong Gu, Yan Yuan, Xuezhong Liu, Guoqiang Zhu, Jianfa Bai, Jianchun Bian, ZongPing Liu

**Affiliations:** 1 College of Veterinary Medicine, Yangzhou University, Yangzhou, 225009 Jiangsu China; 2 Jiangsu Co-innovation Center for Prevention and Control of Important Animal Infectious Diseases and Zoonoses, Yangzhou, 225009 Jiangsu China; 3 Joint International Research Laboratory of Agriculture and Agri-Product Safety, the Ministry of Education of China, Yangzhou University, Yangzhou, 225009 China; 4 Kansas State Veterinary Diagnostic Laboratory, Kansas State University, 1800 Denison Avenue, Manhattan, KS 66506 United States

## Abstract

The aim of this study was to investigate the molecular mechanisms of the destruction of cytoskeletal structure by Zearalenone (ZEA) in mouse-derived TM4 cells. In order to investigate the role of autophagy, oxidative stress and endoplasmic reticulum(ER) stress in the process of destruction of cytoskeletal structure, the effects of ZEA on the cell viability, cytoskeletal structure, autophagy, oxidative stress, ER stress, MAPK and PI3K- AKT- mTOR signaling pathways were studied. The data demonstrated that ZEA damaged the cytoskeletal structure through the induction of autophagy that leads to the alteration of cytoskeletal structure via elevated oxidative stress. Our results further showed that the autophagy was stimulated by ZEA through PI3K-AKT-mTOR and MAPK signaling pathways in TM4 cells. In addition, ZEA also induced the ER stress which was involved in the induction of the autophagy through inhibiting the ERK signal pathway to suppress the phosphorylation of mTOR. ER stress was involved in the damage of cytoskeletal structure through induction of autophagy by producing ROS. Taken together, this study revealed that ZEA altered the cytoskeletal structure via oxidative stress - autophagy- ER stress pathway in mouse TM4 Sertoli cells.

## Introduction

Zearalenone (ZEA) is a mycotoxin from *Fusarium* species commonly found in many food commodities and known to exert estrogenic activities which can cause reproductive dysfunction^1–3^. Several studies have suggested that the exposure to ZEA can reduce the number of germ cells, alter the morphology of testis, cause testicular cells to differentiate abnormally and affect fertility^4^. Many studies showed that the treatment with ZEA can activate cell death, cell autophagy and cell apoptosis in Leydig cells^5–7^. Additionally, a series of recent publications have revealed that ZEA induced cell death of RAW 264.7 macrophages through ER stress, and application of a herb inhibited the ZEA-induced cell death^8,9^. The toxicity of ZEA and its metabolites is not only due to the previously mentioned estrogenic effect, but other mechanisms such as oxidative stress and DNA damage may be involved^10^. Several studies have shown that the oxidative stress may play a major role in the cytotoxic effects of ZEA and its metabolites^11,12^.

The cytoskeleton, composed primarily of actin microfilaments, intermediate filaments, and microtubules, is considered an important mediator of mechanical forces in individual cells^13^. Actin filaments, being most abundant among all cytoskeletal constituents, make the largest contribution to the mechanical properties of cell. Cells often modify their actin filamentous network in response to biochemical changes^14^. Recent publications have revealed that the biogenesis and trafficking of autophagy depend on the activities of several cytoskeletal components including actin assembly factors, signaling proteins and microtubule or actin-based motors^15,16^.

Sertoli cells around germ cells are considered a barrier that protects spermatogenesis from harmful influences^17^. One of the major roles of Sertoli cells is to establish the blood-testis barrier (BTB) which provides an exclusive and stable environment for germ cell development^18^. BTB is a highly organized junctional complex composed of a series of tight junctions, actin-based adherens junction, intermediate filament based desmosome like junctions and gap junctions^19^. Thus, any agent that can impair the stability or function of Sertoli cells may profoundly affect spermatogenesis^18^. The TM4 cell line was derived from the mouse Sertoli cells. It can provide a useful model for testing the male reproductive toxicity and the underlying mechanism^20,21^. Thus, in current study, the TM 4 cell line was selected as the experimental subject for exploring the reproductive toxicity of ZEA.

Our previous study has shown that ZEA can disrupt the cytoskeletal structure and ultrastructure in Sertoli cells^22^. However, the underlying molecular mechanism of the cytotoxicity is unclear. Thus, the purpose of the present study was to determine the possible mechanism by which ZEA destroyed the cytoskeletal structure. For this purpose, the role of oxidative stress, autophagy, and ER stress were investigated, and the relationships among them were also studied in TM4 cells that exposed to ZEA.

## Results

### Analysis of the cell viability

**Figure 1:**
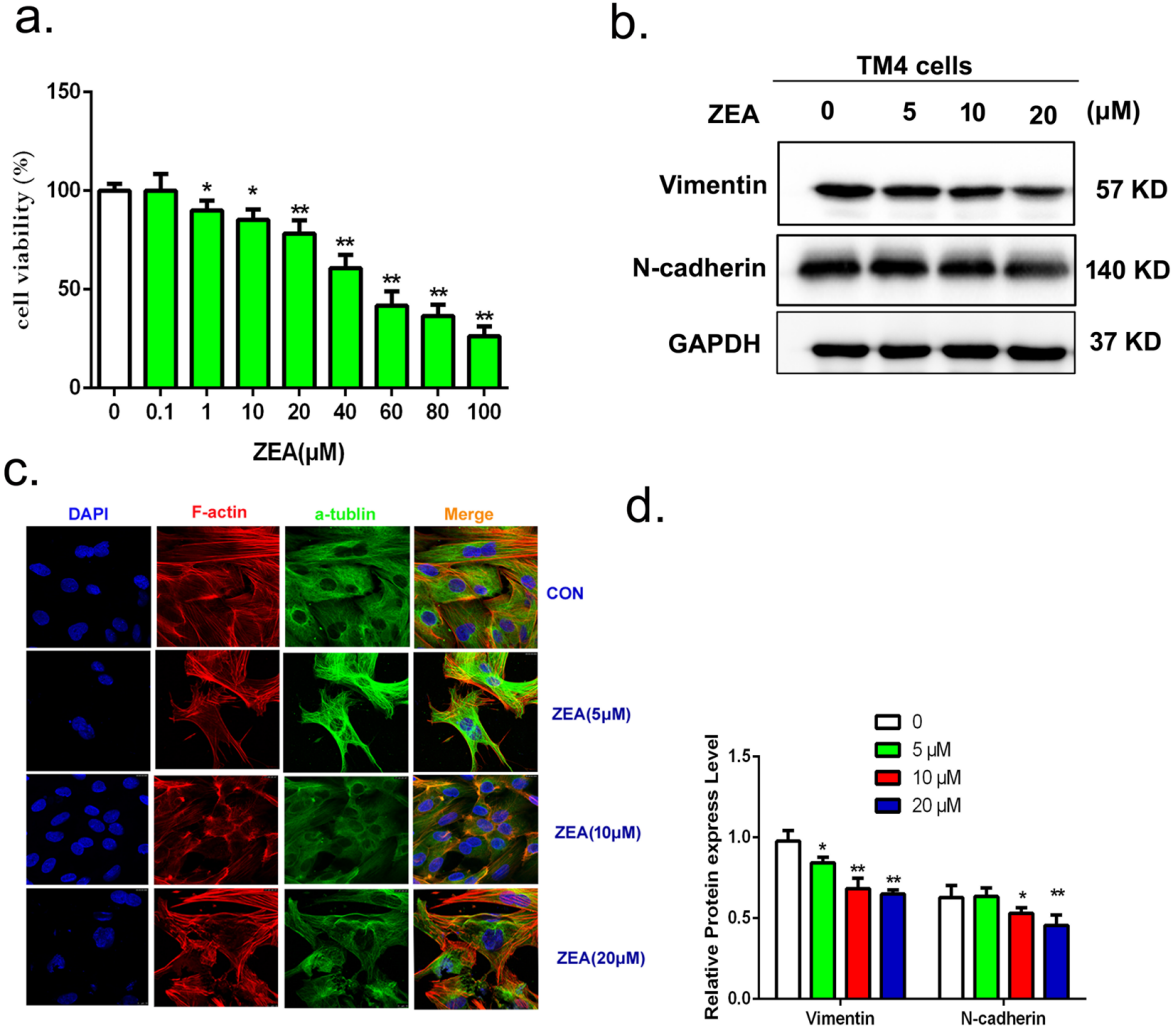
ZEA inhibits TM4 cells proliferation and damages the cytoskeletal structure. (**a**) Cell viability analysis of TM4 cells using a CCK8 assay kit. (**b**) The expressions of N-cadherin and vimentin were analyzed by using western blotting. (**c**) Confocal immunohistochemistry analysis of α-tubulin, F-actin, and nucleus structure in TM4 cells. The green, red and blue fluorescence indicate the α-tubulin, F-actin, nuclei respectively. (**d**) Blots for N-cadherin and Vimentin were semi-quantified using Image Lab. Values represent the mean ± S.D. *P < 0.05, **P < 0.01 versus the control group.

Cell viability was examined by using the cell counting kit-8 (CCK8) assay and 50% inhibitive concentration (IC50) of ZEA was examined also. After the TM4 cells were treated by different concentrations of ZEA for 24 h, the cell viability was assessed by the cell counting kit-8 (CCK8) assay. The results showed that after the TM4 cells were treated by different concentrations of ZEA for 24 h, the viability of the TM4 cells were decreased in a dose-dependent manner (Fig. 1a). The survival rates of TM4 cells were more than 73.03% in ZEA treatment groups at equal or lower than 30 μM. However, the survival rate was 53.35% in the 50 μM group, which was close to the IC50. Thus in this study, we selected the 0.1, 1, 5, 10, 20 and 30 μM ZEA as the treatment concentrations.

### ZEA damaged the cytoskeletal structure and affected the expressions of cytoskeleton associated proteins in TM4 cells

The changes by ZEA on the cytoskeletal structure were detected by using confocal laser-scanning microscope. As is shown in the (Fig. 1c), the blue, green and red fluorescence respectively indicate the nucleus, α-tubulin and F-actin of the cells. The cells in the control group exhibited well-organized actin filaments and the α-tubulin microtubules. However, after 24 h exposure to ZEA, the F-actin filaments and α-tubulin microtubules were destroyed. This disruption was more serious with the increasing dosages. The results from western blotting showed that compared with the control group, the expressions of vimentin and N-cadherin decreased significantly after treatment with different concentration of ZEA (Fig. 1b and d). These data indicated that ZEA can damage the cytoskeletal structure and reduce the expressions of vimentin and N-cadherin.

### ZEA destroyed the cytoskeletal structure through the induction of autophagy in TM4 cells

**Figure 2:**
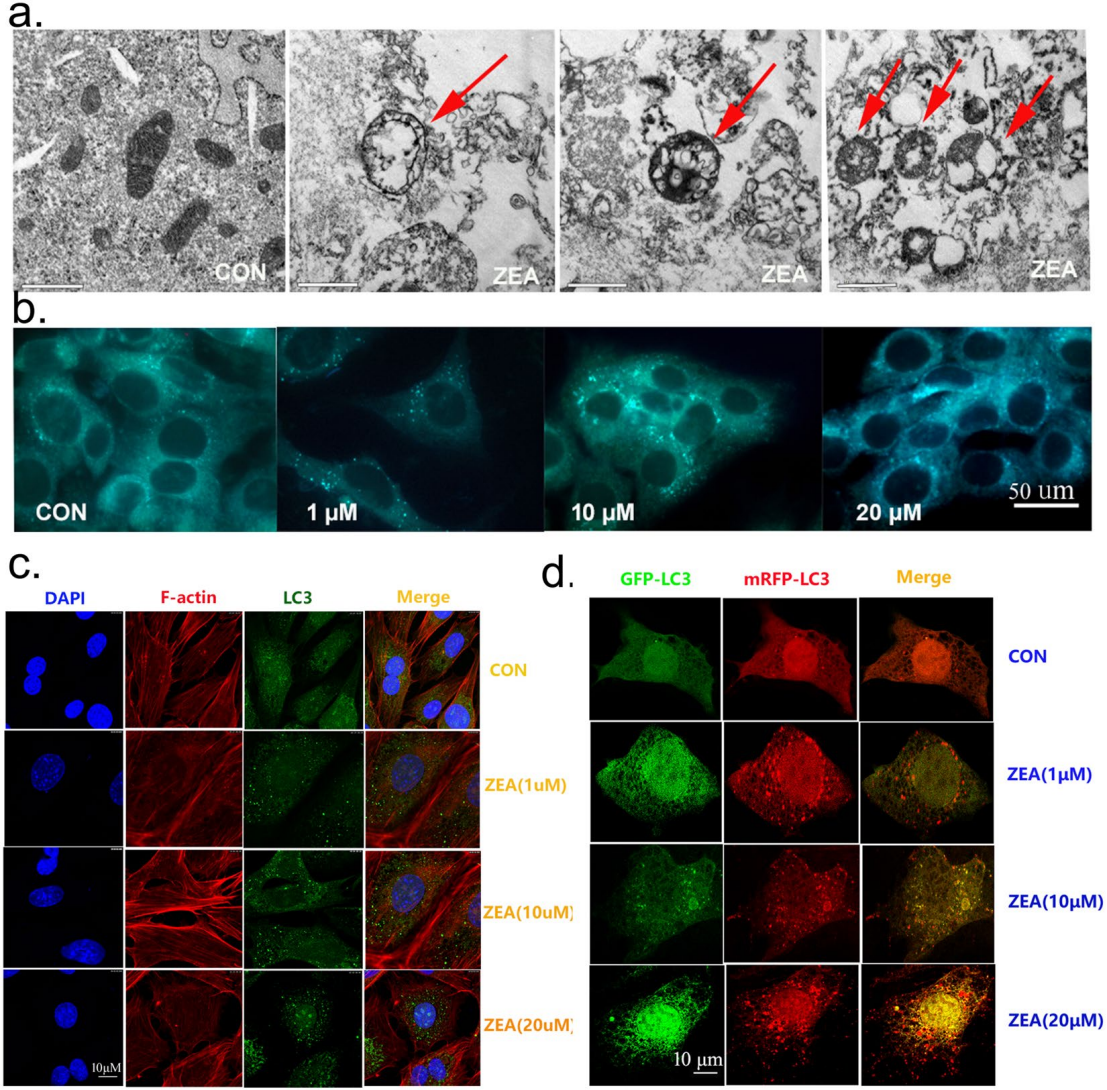
ZEA can stimulate autophagy in TM4 cells. (**a**) Red arrows indicate autophagic vacuoles observed by TEM. (**b**) The detection of autophagic vacuoles by using MDC (100×). (**c**) LC-3 puncta were observed by fluorescence microscopy (630×). (**d**) Exposure to ZEA caused pronounced formation of LC3 puncta that displayed both green and red fluorescence producing a yellow overlay.

**Figure 3:**
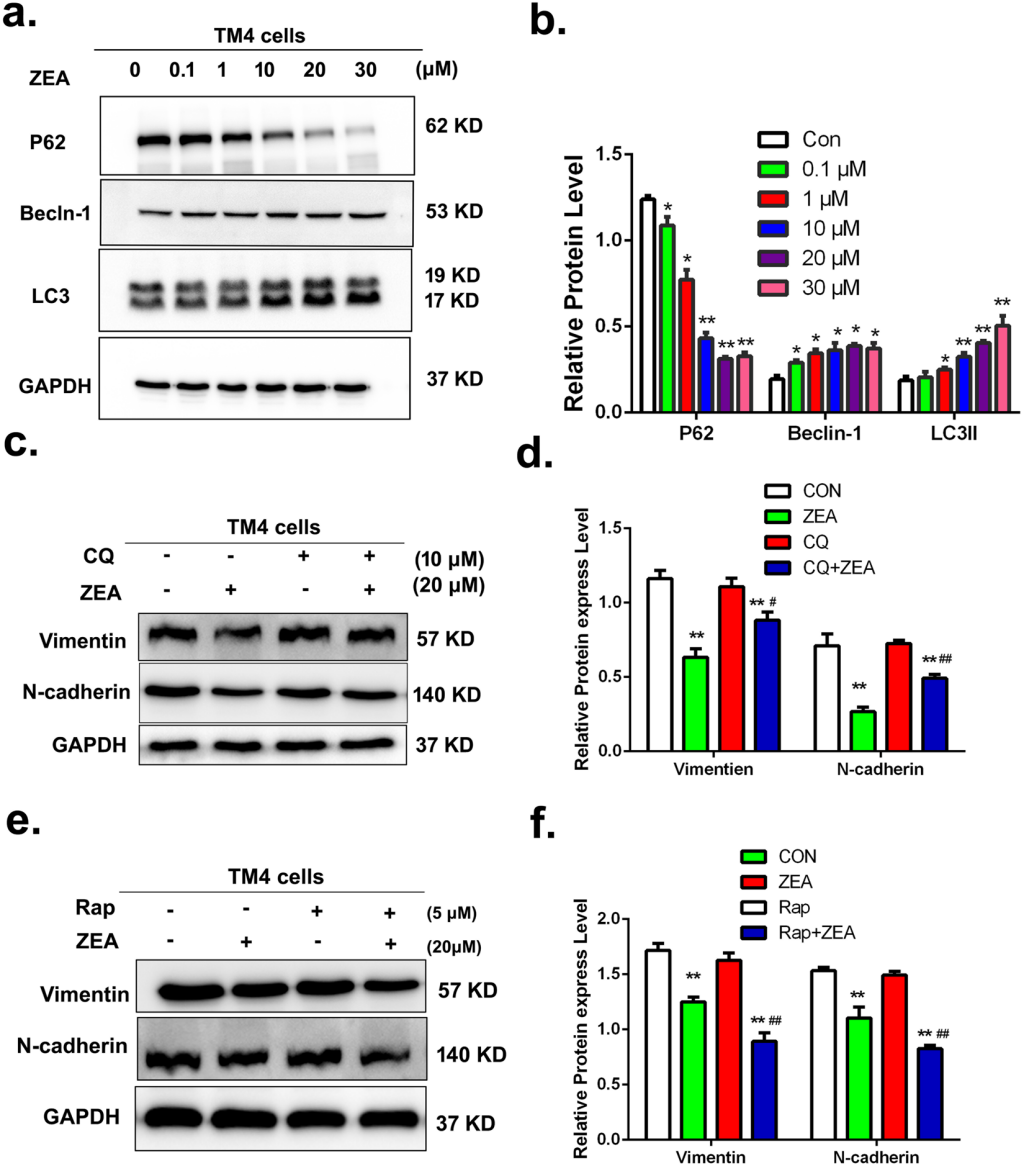
ZEA destroyed the cytoskeletal structure through causing autophagy. (**a**,**b**) The expressions of LC3, P62 and Beclin-1were were analyzed by using western blotting. (**c**–**f**) TM4 cells were pretreated with 10 μM CQ or 5 μM Rap for 30 min and then treated with ZEA for an additional 24 h. Cells were harvested and subjected to western blotting with an antibody against N-cadherin and Vimentin. Values represent the mean ± S.D. *P < 0.05, **P < 0.01 versus the control group; ^#^P < 0.05, ^##^P < 0.01 versus the 20 μM ZEA group.

To determine the role of autophagy in ZEA induced damage of the cytoskeletal structure, we analyzed the effects of ZEA on autophagy in the TM4 cells. The results from TEM showed that ZEA stimulated the formation of lysosomes as well as autophagosomes in TM4 cells (Fig. 2a). To further explore the effect of ZEA on autophagy, we studied the formation of autophagosomes by using Monodansylcadaverine (MDC) staining. The results showed that the number of MDC foci was significantly increased in a dose-dependent manner after treatment with ZEA for 24 h (Fig. 2b). The change of endogenous LC3 distribution was analyzed by confocal immunohistochemistry. As shown in Fig. 2c, after group, the ZEA-treated groups showed a remarkable increase of LC3-positive puncta as compared to the control. In addition, we also detected the change of LC3 puncta by transfecting the EGFP-mRFP-LC3B adenovirus. As shown in Fig. 2d, the formations of LC3 puncta, as assessed by EGFP-LC3 and mRFP-LC3 colocalization, were increased significantly in the treated groups. Finally, the data from western blotting showed that the expressions of LC3-II and Beclin-1 were increased significantly in a concentration-dependent manner after the 24 h ZEA treatment (Fig. 3a and b). The expression of the P62 protein was significantly decreased after the treatment with ZEA compared to the control group. These data suggested that ZEA can trigger the autophagy in TM4 cells.

**Figure 4:**
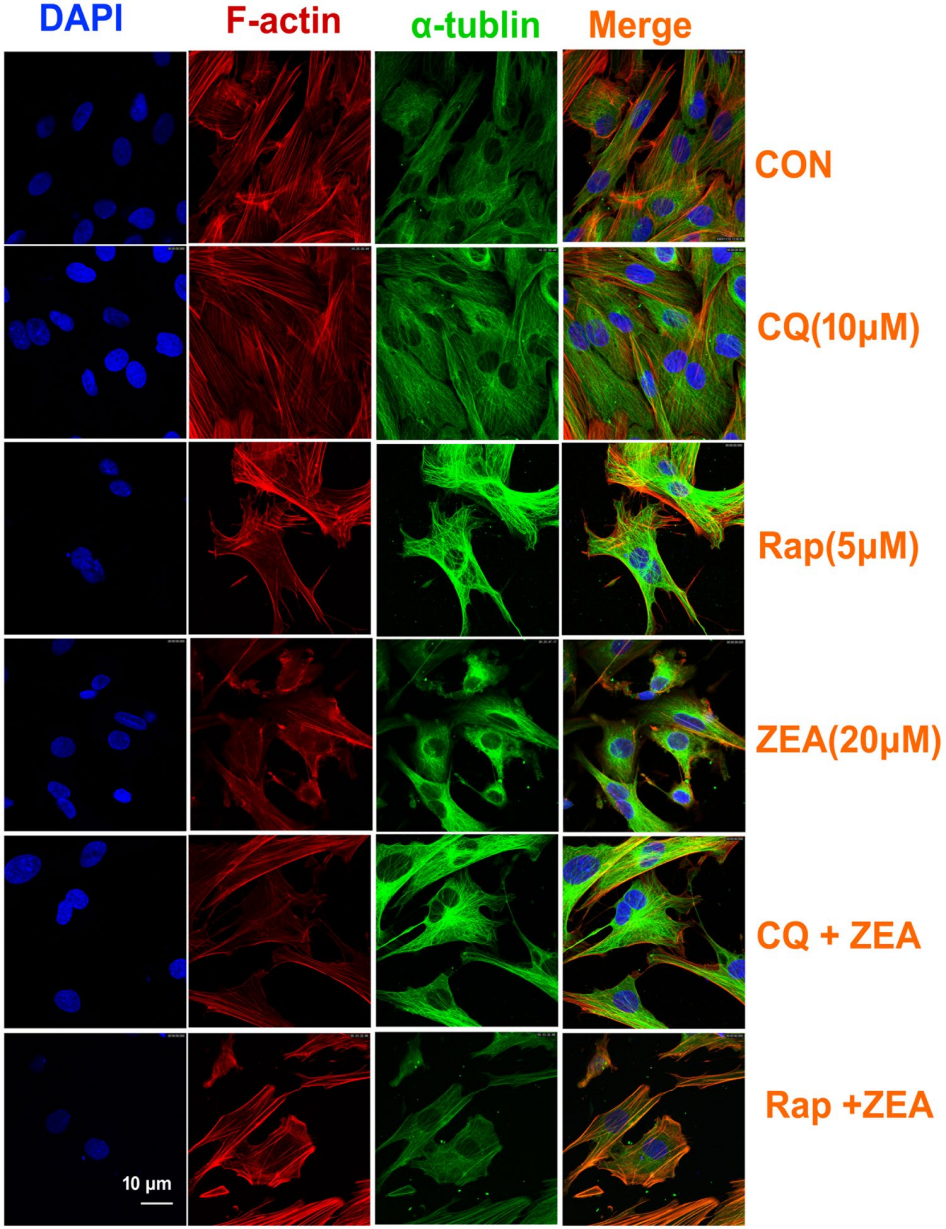
Autophagy mediated the damage of the cytoskeletal structure. TM4 cells were pretreated with 10 μM CQ or 5 μM Rap for 30 min and then treated with ZEA for an additional 24 h. Confocal immunohistochemistry analysis of α-tubulin, F-actin, and nucleus structure in TM4 cells. The green and red fluorescence indicate the α-tubulin and F-actin, respectively. The nuclei were stained in blue using DAPI.

In order to understand the role of ZEA on impaired cytoskeletal structure and autophagy, the autophagic inhibitor chloroquine (CQ) and inducer rapamycin (RAP) were introduced into this study. The western blot data showed that compared to the ZEA alone group, the expressions of vimentin and N-cadherin were significantly increased in the co-treatment of ZEA and CQ group, while, the expressions of vimentin and N-cadherin were significantly decreased in the co-treatment of RAP and ZEA group compared to the ZEA alone group (Fig. 3c–f). What’s more is that the data from confocal immunohistochemistry suggested that the disruption of α-tubulin and F-actin was alleviated in the group of co-treatment with CQ and ZEA as compared to the control group (Fig. 4). However the damage of α-tubulin and F-actin was aggravated after co-treatment with RAP and ZEA. Taken together, these data showed that ZEA can induce autophagy in TM4 cells and the autophagy was involved in the process of destroying the cytoskeletal structure.

### Autophagy impaired the cytoskeletal structure via causing oxidative stress in the TM4 cells exposure to ZEA

**Figure 5:**
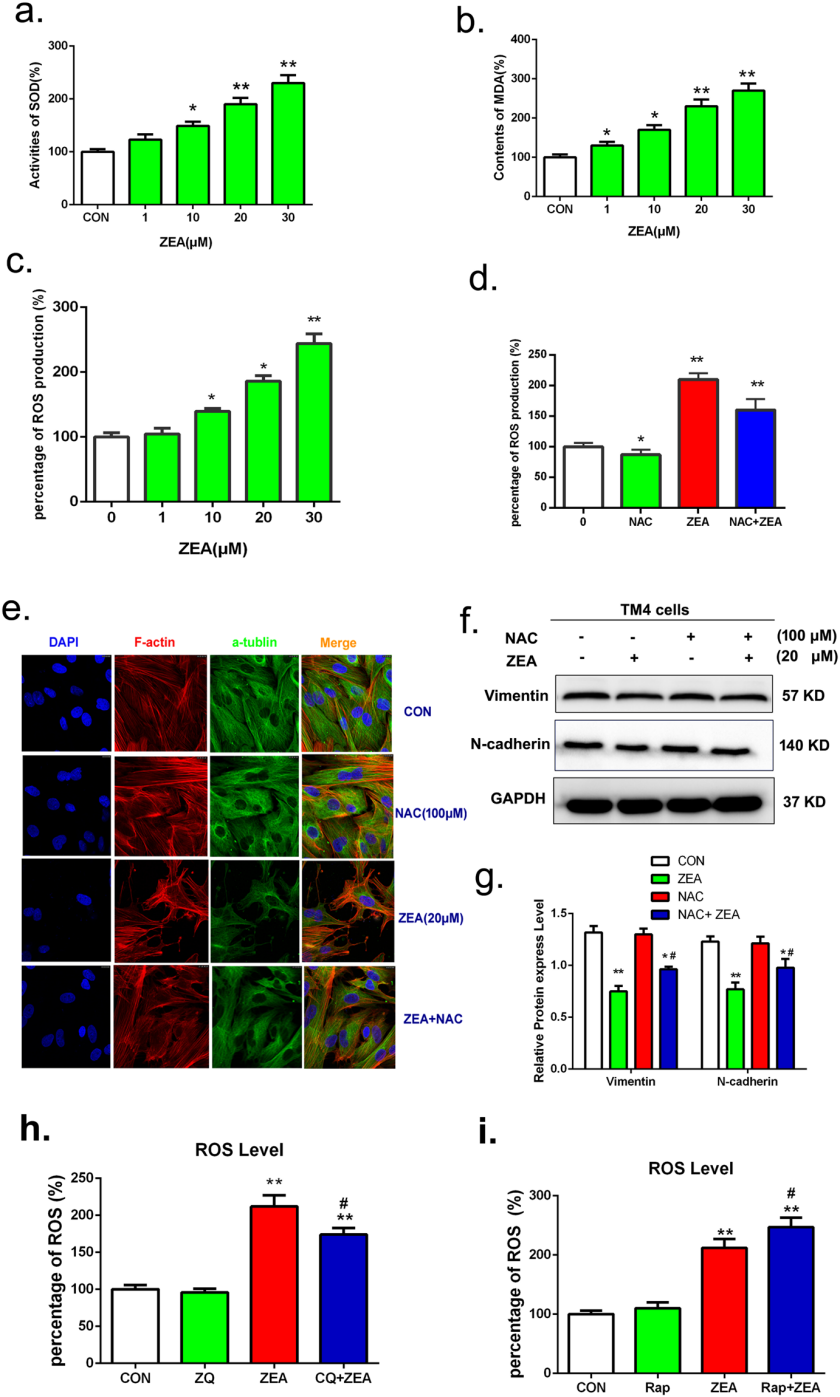
Autophagy impaired the cytoskeletal structure via causing oxidative. (**a**–**c**) ZEA induced the oxidative stress of TM4 cell (**d**) NAC can protect the overproduction of ROS induced by ZEA. (**e**) NAC treatment could alleviate the disruption of the cytoskeletal. (**f**,**g**) The expressions of N-cadherin and Vimentin were detected by using western blotting. (**h**,**i**) The level of ROS were detected by using the flow cytometry. Values represent the mean ± S.D. *P < 0.05, **P < 0.01 versus the control group; ^#^P < 0.05, ^##^P < 0.01 versus the 20 μM ZEA group.

In order to explore the molecular mechanisms of autophagy in destroying the cytoskeletal structure, we investigated the effects of ZEA on oxidative stress. As shown in Fig. 5a,b and c, compared to the control group, the levels of Superoxide dismutase (SOD), Malondialdehyde (MDA) and reactive oxygen species (ROS) were increased gradually in a dose-dependent manner. While, after co-treatment with the antioxidant NAC, the intracellular level of ROS was significantly decreased compared to the cells treated with ZEA alone (Fig. 5d). The data from confocal immunohistochemistry showed that compared with the ZEA alone group, NAC treatment could alleviate the disruption of the cytoskeletal structure (Fig. 5e). The data from western blot showed (Fig. 2f and g) that the expressions of vimentin and N-cadherin were increased significantly in the co-treatment with NAC and ZEA group compared to the ZEA alone group.

In addition, the relationship between autophagy and ROS was detecting by using the autophagic inhibitor CQ and inducer rapamycin RAP. The results suggested (Fig. 5h and i) that compared to the ZEA alone group, the level of ROS was decreased in the group of co-treatment with CQ and ZEA, while the level of ROS was increased in the group of co-treatment with RAP and ZEA. These results suggested that ZEA can cause oxidative stress which can destroy the cytoskeletal structure and the oxidative stress was mediated by autophagy in TM4 cells.

### The autophagy was stimulated by ZEA through PI3K-AKT-mTOR and MAPK signaling pathways in TM4 cells

**Figure 6:**
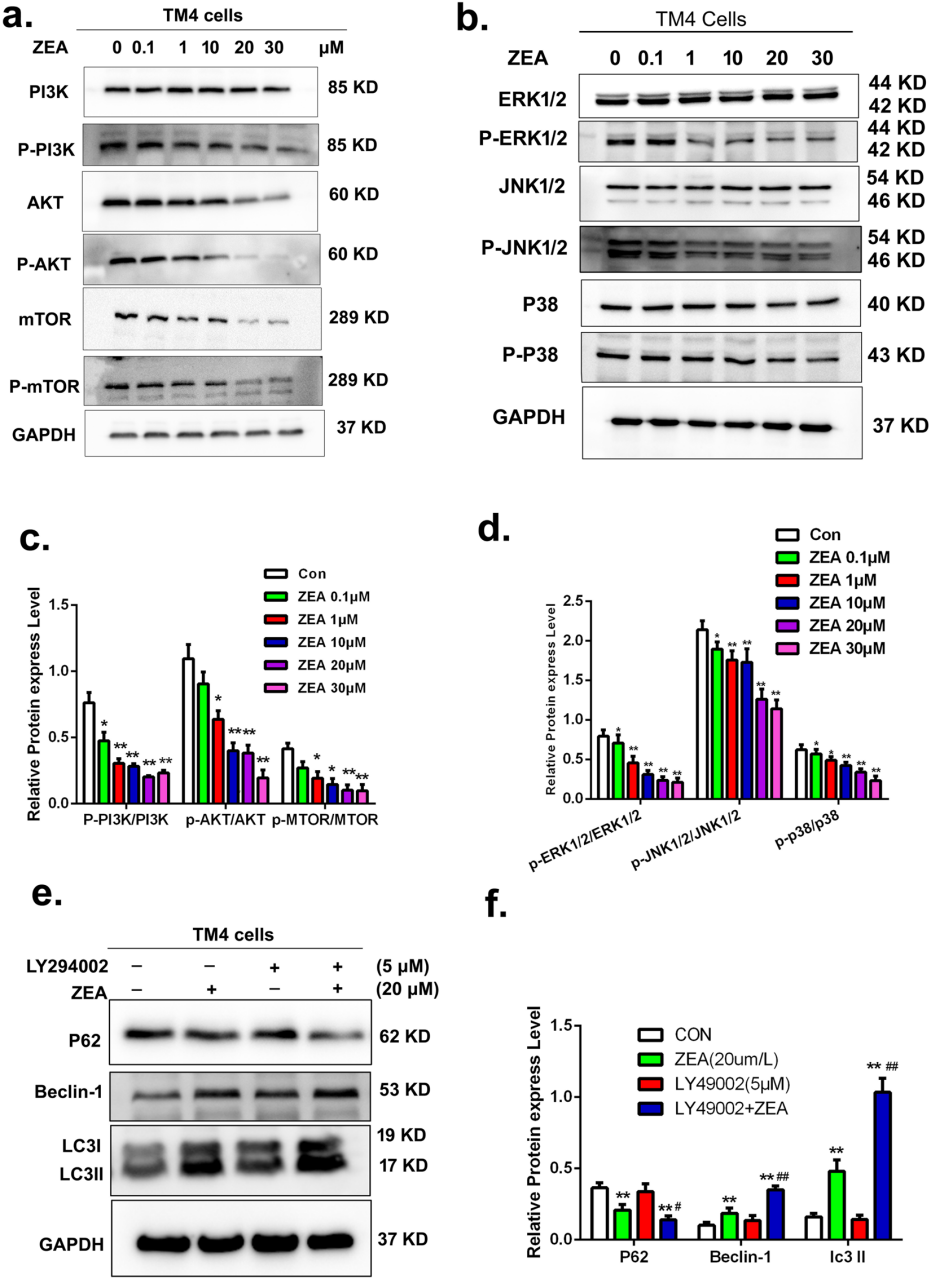
The effects of ZEA on the MAPK family proteins and PI3K-AKT-mTOR pathway. (**a**,**c**) The expressionf of p-PI3K, PI3K, p-AKT, AKT, p-mTOR and mTOR.were detected by western blotting. (**b**,**d**) The expression of MAPK family proteins were detected by using western blotting. (**e**,**f**) After adding LY294002, the expressions of LC3II, P62 and Beclin-1 were detected by using Western blotting. Values represent the mean ± S.D. *P < 0.05, **P < 0.01 versus the control group; ^#^P < 0.05, ^##^P < 0.01 versus the 20 μM ZEA group.

To investigate the mechanisms of ZEA induced autophagy, we monitored the effects of ZEA on the PI3K-AKT-mTOR and MAPK signaling pathways. The results from western blot suggested (Fig. 6a and c) that compared with the control group, the ratios of p-PI3K/PI3K, p-AKT/AKT and P-mTOR/mTOR were significantly decreased after the treatment with ZEA. Similarly, the ratios of MAPK family proteins p-ERK1/2/ERK1/2, p-JNK1/2/JNK1/2 and p-p-38/P38 were also significantly decreased in a concentration dependent manner (Fig. 6b and d).

To further explore the role of the PI3K-Akt-mTOR signal pathway in ZEA induced autophagy, the PI3K inhibitor, LY294002, was used to inhibit the PI3K-Akt signal pathway. The results from western blot showed that the expressions of LC3II and Beclin-1 proteins were significantly decreased in cells co-treated with LY294002 and ZEA compared with the cells exposed to ZEA alone (Fig. 6e and f). The expression of the P62 protein was significantly increased compared to the ZEA treatment. The data indicated that the PI3K-Akt-mTOR signal pathway was involved in the ZEA induced autophagy.

**Figure 7:**
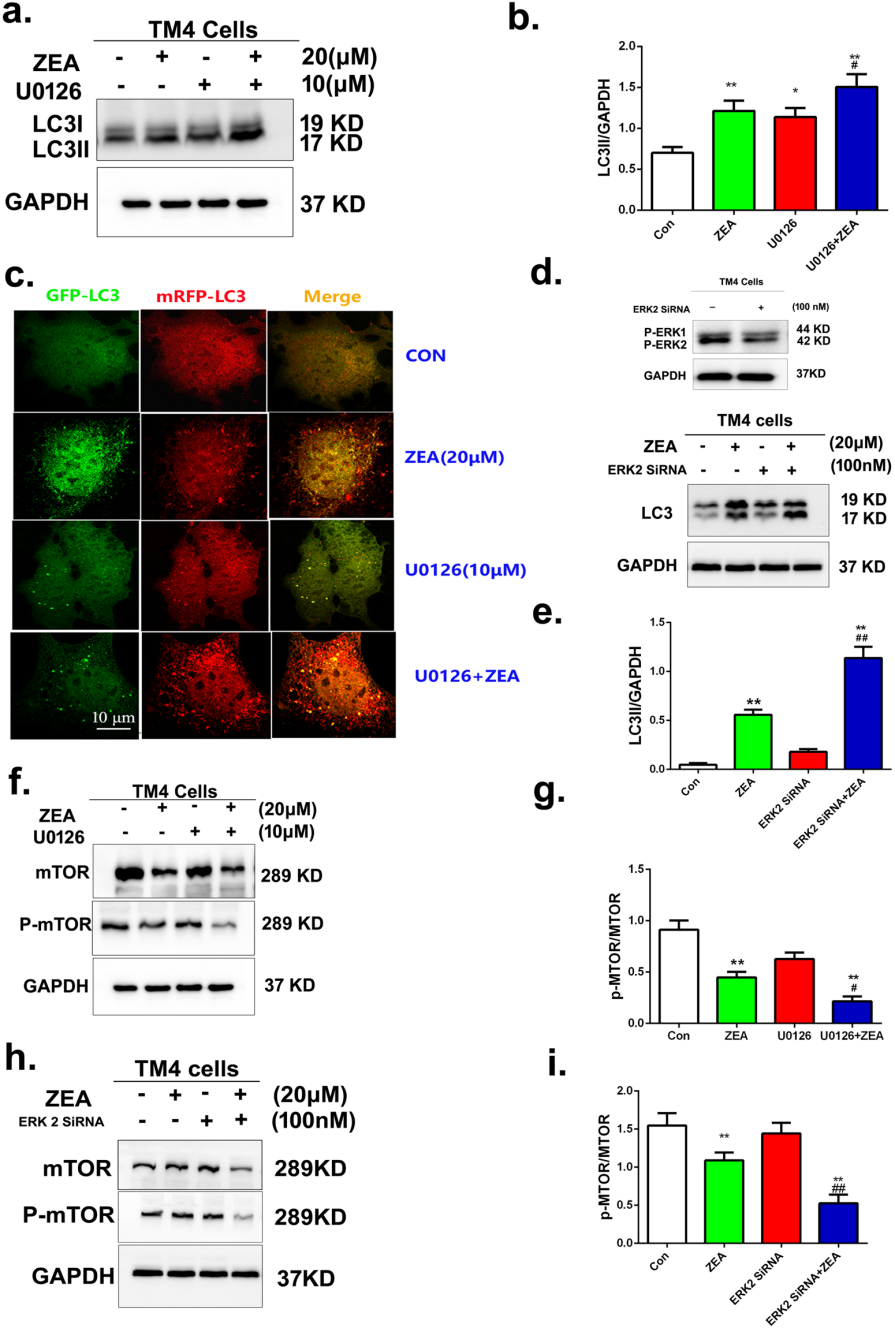
The ERK1/2 pathway was participated in the induction of autophagy. (**a**,**b**) The expression of LC3was detected by using western blotting. (**c**) The EGFP-LC3 and mRFP-LC3 colocalization was detected by confocal immunohistochemistry. (**d**,**e**) The expression of LC3 were detected by using western blotting. (**f**–**i**) Western blotting analysis was performed to detect the expressions of p-mTOR and mTOR. Values represent the mean ± S.D. *P < 0.05, **P < 0.01 versus the control group; ^#^P < 0.05, ^##^P < 0.01 versus the 20 μM ZEA group.

In order to determine the role of ERK pathway in the ZEA induced autophagy, we used the U0126 to inhibit the phosphorylation of ERK and SiRNA of ERK2 to silence the ERK2 expression. After treatment with U0126 and siRNA of ERK2 the level of autophagy was increased. The data from western blot showed that the levels of LC3II proteins significantly increased in cells co-treated with U0126 and ZEA compared to the cells exposed to ZEA alone (Fig. 7a and b). The results from confocal immunohistochemistry suggested that the formation of LC3 puncta was significantly increased in the cells co-treated with U0126 and ZEA compared with the cells exposed to ZEA alone (Fig. 7c). As expected, after silencing the expression of ERK 2 using ERK2 siRNA, the expression of P-ERK2 was remarkably decreased and the level of LC3II protein was significantly increased in cells co-treated with the ERK2 siRNA and ZEA compared to the cells exposed to ZEA alone (Fig. 7d and e).

Additionally, the p-mTOR/mTOR ratio was significantly decreased in the cells co-treated with U0126 and ZEA or the cells co-treated with the ERK2 siRNA and ZEA compared to the cells exposed to ZEA alone respectively (Fig. 7f–i). Collectively, these data indicated that the PI3K-Akt-mTOR and ERK signaling pathways were involved in the process of ZEA induced autophagy. ZEA triggered the autophagy in TM4 cells through the ERK signaling pathway to suppress the phosphorylation of mTOR.

### ER stress was involved in the induction of autophagy via inhibiting the ERK signal way to suppress the phosphorylation of mTOR

**Figure 8:**
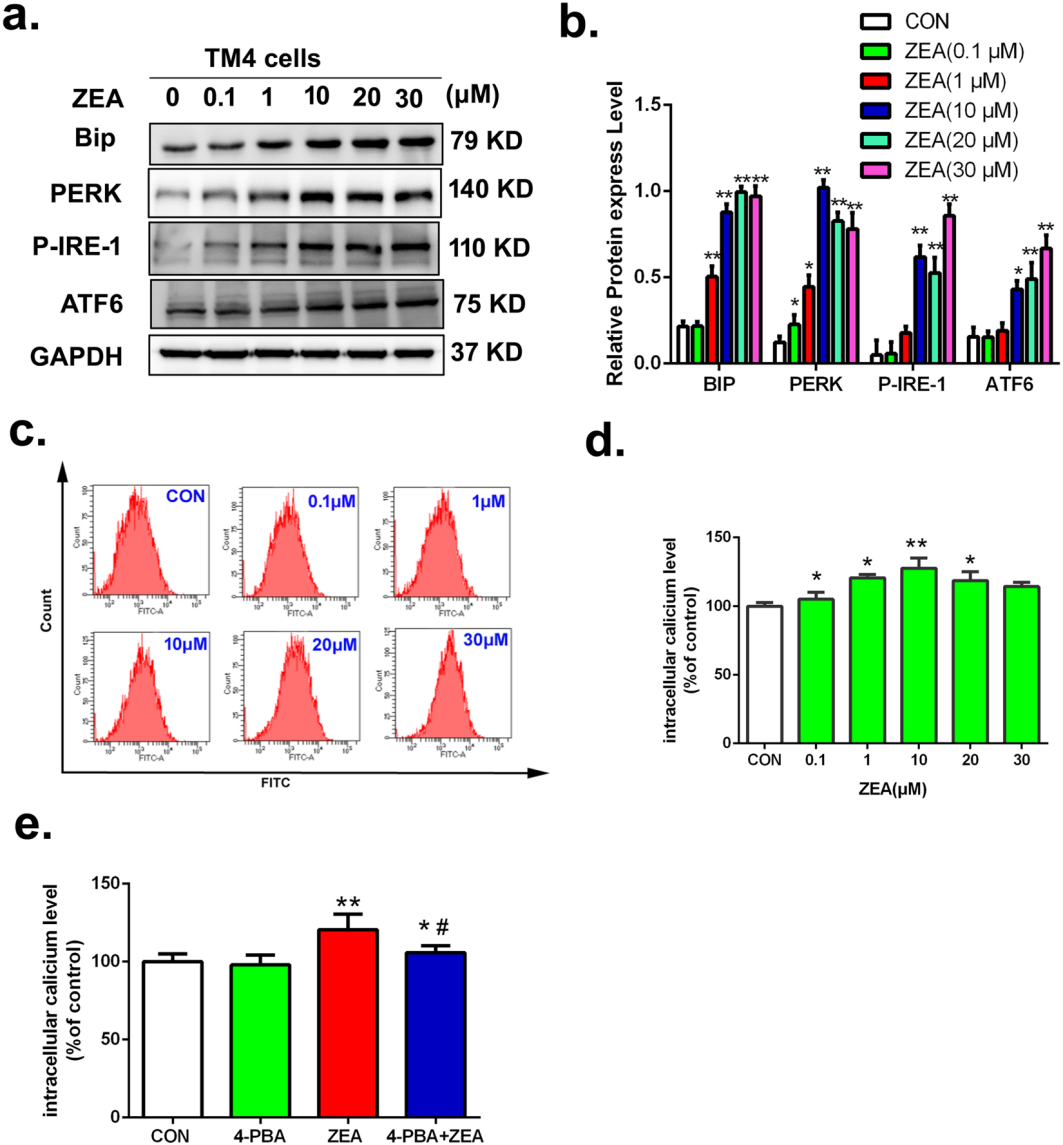
ZEA induced endoplasmic reticulum stress in TM4 cells. (**a**,**b**) Cells were harvested and subjected to western blotting with antibodies against BiP, PERK,P-IRE1 and ATF6. (**c**,**d**) The intracellular was examined by using the Fluo-3 AM indicator. (**e**) TM4 cells were co-treated with 1 mM 4-PBA for 1 h and then treated with 20 μM ZEA for an additional 24 h. The intracellular was examined using the Fluo-3 AM indicator and flow cytometry analysis. Values represent the mean ± S.D. *P < 0.05, **P < 0.01 versus the control group; ^#^P < 0.05, ^##^P < 0.01 versus the 20 μM ZEA group.

Our results indicated that ZEA induced autophagy partially via its inhibition of the ERK and PI3K-AKT-mTOR signaling pathways. However, the mechanisms of ZEA inhibition on ERK and PI3K-AKT-mTOR signaling ways were unclear. Thus, we investigated whether ER stress was involved in the suppression of ERK in TM4 cells. This study examined the expression patterns of several molecular indicators of ER stress. The results from western blot suggested that the expressions of BiP, PERK, P-IRE1and ATF6 proteins were significantly increased in the ZEA-treatment group compared to the control group (Fig. 8a and b). The flow cytometry data demonstrated (Fig. 8c and d) that compared with the control group the level of intracellular Ca^2+^ was remarkably increased in the ZEA-treated groups. Taken together, these data suggested that ZEA can induce ER stress in TM4 cells.

**Figure 9:**
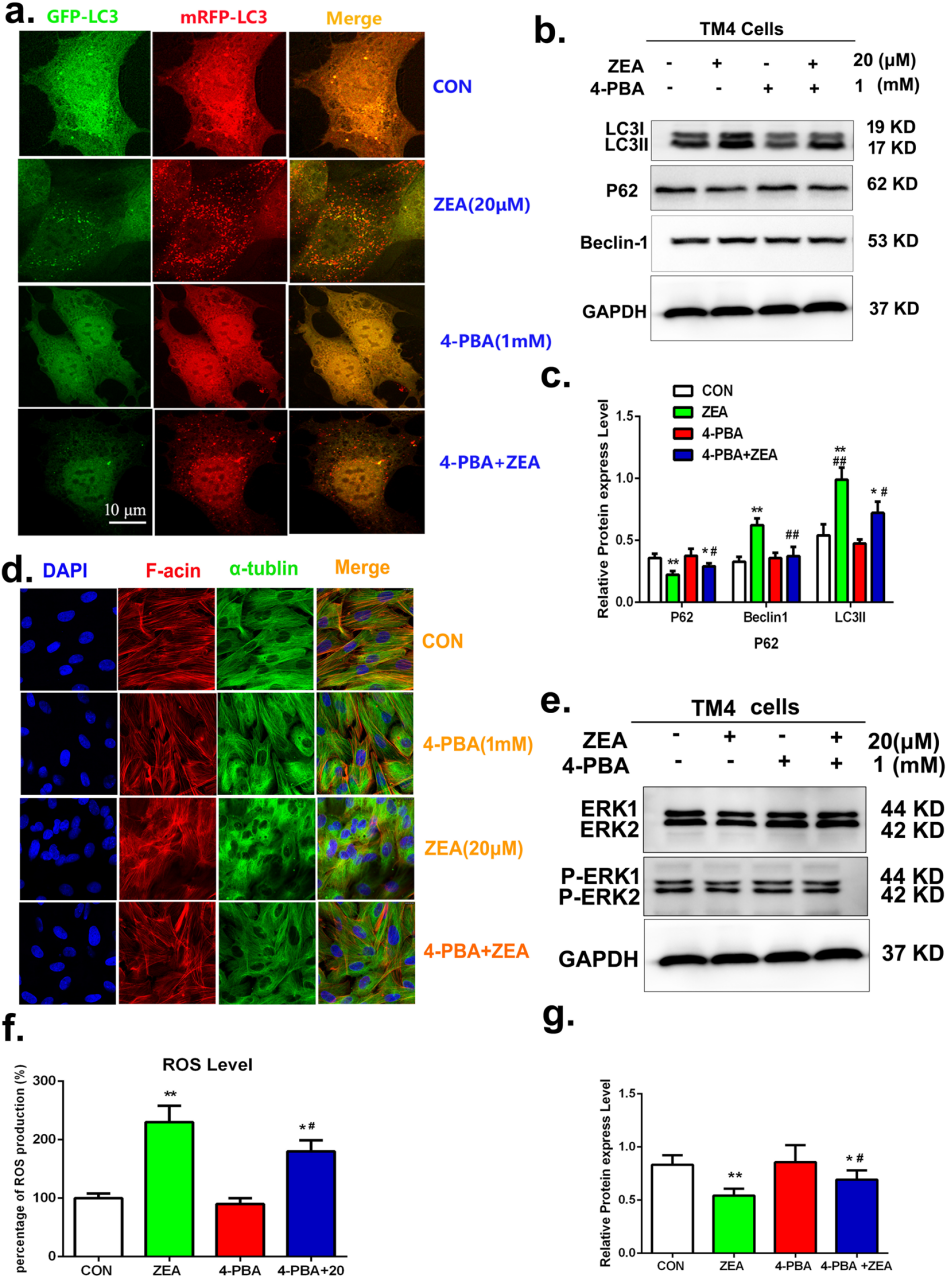
ER stress was involved in the damage of cytoskeletal structure. (**a**) The formation of LC3 puncta was detected using confocal immunohistochemistry. (**b**,**c**) The expressions of P-62, Beclin-1, and LC3by using the western blotting. (**d**) Confocal immunohistochemistry analysis of α-tubulin, F-actin, and nucleus structure. (**e**,**g**) Western blotting was performed to detect the expression of ERK1/2 and P-ERK1/2. (**f**) The level of ROS were detected by using Flow cytometry. Values represent the mean ± S.D. *P < 0.05, **P < 0.01 versus the control group; ^#^P < 0.05, ^##^P < 0.01 versus the 20 μM ZEA group.

To further investigate the role of ER stress in ZEA induced autophagy, 4-phenyl butyric acid (4-PBA), which is a protein chaperone that stabilizes unfolded proteins and facilitates their proper folding, was used to alleviate ER stress signals. TM4 cells were pretreated with 1 mM 4-PBA for 1 h and then treated with 20 μM ZEA for an additional 24 h. As shown in Fig. 8e, the level of intracellular Ca^2+^ was significantly decreased in the 4PBA pre-treated cells compared with cells treated with ZEA alone. The data from cells transfected with EGFP-mRFP-LC3B adenovirus demonstrated that the formation of LC3 was significantly decreased in cells co-treated with 4-PBA and ZEA compared with cells treated with ZEA alone (Fig. 9a). Figure 9b and c showed that the levels of LC3II and Beclin-1 proteins were significantly decreased in cells co-treated with 4-PBA and ZEA compared to the cells exposed to ZEA treatment alone. The expression of P62 was significantly increased after the co-treatment of 4-PBA and ZEA. These data showed that the ER stress was involved in the ZEA induced autophagy.

Additionally, the ratio of p-ERK1/2/ERK1/2 was significantly increased in cells co-treated with 4-PBA and ZEA compared with the cells exposed to ZEA alone (Fig. 9e and g). Taken together, these data suggested that The ER stress stimulated autophagy via inhibiting the ERK signal way to suppress the phosphorylation of mTOR.

### ER stress was involved in the damage of cytoskeletal structure through causing autophagy to produce the ROS

The data from flow cytometry showed that after pre-treatment with the 4-PBA to down regulate the ER stress signal, the intracelluar ROS content was decreased significantly compared with cells treated with ZEA alone (Fig. 9f). The data from confocal immunohistochemistry showed that pre-treatment with 4-PBA can partially alleviate the disruption of the cytoskeletal compared with the ZEA alone group (Fig. 9d). These data suggested that ER stress was involved in the damage of cytoskeletal structure through causing autophagy to produce the ROS

## Discussion

Autophagy was triggered as the degradation system and dynamic recycling system in cells and cytoplasmic materials were transported and delivered for degradation within the double-membrane vacuoles inside lysosomes^23^. Under normal physiological conditions, the cell maintained a low level of autophagy. While, during various stress conditions such as oxidative stress, endoplasmic reticulum stress and nutrient limitation the autophagy will be triggered for survival^24^. But, accumulating evidence showed that the aberrant and excessive action of autophagy could destroy normal proteins and organelles leading to cell death which is generally termed type-II programmed cell death^24,25^. In current study, the potential rationale for why autophagy was involved in destruction of the cytoskeletal structure is that the aberrant and excessive autophagy was induced by ZEA in TM4 cells.

Recently, the themes of oxidative stress and autophagy have been brought together^26^. Several studies showed that the ZEA treatment can activate autophagy and oxidative stress in different cells^6,12^. What’s more is that the increasing evidence demonstrated that the formation of ROS has been implicated in disruption of microtubules^27–29^. Study has suggested that oxidative stress decreases microtubule growth and stability in ventricular myocytes^30^. In addition, hydrogen peroxide treatment can depolymerize and disorganize the microtubules in AR42J pancreatic epithelial cells^31^. Thus, it is speculated that ROS was involved in the process of alteration of cytoskeletal structure through autophagy. The current study revealed that autophagy impaired the cytoskeletal structure via causing oxidative stress in the TM4 cells upon the exposure to ZEA.

Autophagy can be triggered through multiple interconnected pathways, such as the MAPK, PI3K-AKT-mTOR and ATP-AMPK-mTOR signaling pathways^32,33^. MAPK signaling pathways have a vital role in interfering with autophagy in several types of cells^34^. The mTOR protein is a master modulator that involved in the induction of autophagy. Up-regulating the mTOR pathway can decrease the level of autophagy, whereas down-regulating the phosphorylation of mTOR can increase the level of autophagy^35^. The PI3K-Akt-mTOR signaling pathway is a major signal transduction cascade involved in cellular metabolism, proliferation, and survival, and plays an important role in autophagy^36^. The current study suggested that ERK1/2 and PI3K- AKT- mTOR signaling pathways were involved in the induction of autophagy.

Recently, emerging evidences indicated that the ER stress pathway is another important pathway of inducing autophagy^37,38^. The endoplasmic reticulum is a large membrane-enclosed cellular organelle in eukaryotes to precede folding of membrane and secrete proteins. Physiologic and pathological stresses such as aggregate-prone proteins, glucose deprivation, hypoxia, and efflux from the ER can lead to the failure of protein folding and then causing ER stress^37,39^. With the accumulation of unfolded proteins and damaged organelles during ER stress, autophagy can be activated by the unfolded protein response (UPR)^40^. This study showed that ER stress and ERK1/2 signaling pathways were involved in the induction of autophagy in TM4 cells. Recent research has shown that MAPK signaling pathways play a role in ER stress^41^. Thus we expected to find out the relationship between the ER stress and the inhibiting of ERK. Interestingly, our data showed that the ER stress stimulated autophagy via inhibiting the ERK signal way to suppress the phosphorylation of mTOR. These results were similar to the research that ER stress elicits functional autophagy in renal proximal tubular cells via the ERK pathway^42^.

The relationships among ER stress, autophagy and ROS are rather complicated. Several studies showed that ER stress and autophagy were stimulated by the production of ROS^43,44^. However, the increasing number of studies has illustrated that ER stress and autophagy could disrupt the delicate balance between ROS production and degradation^45,46^. ROS accumulation may be caused by either an increase in ROS production or decreased ROS degradation. ER stress results indicated that ROS was generated from the microsomal monooxygenase (MMO) system^45^. The MMO system also leads to the release of large amounts of ROS from the P450 enzyme without substrate oxidation^46^. Study indicated that autophagy can contribute to ROS accumulation by selectively removing catalase, which is a lethal event to the cell^47^. Since the ER stress also mediated the level of autophagy, it was possible that the ER stress regulated the production of ROS through autophagy.

In summary, the current study suggested that ZEA altered the cytoskeletal structure via oxidative stress - autophagy- ER stress pathway in mouse TM4 Sertoli cells.

### Reagents and antibodies

Zearalenone (ZEA) and 4-phenylbutyrate (4-PBA) were purchased from Sigma–Aldrich (St. Louis, MO, USA); Dulbecco’s modified Eagles medium with Hams F-12 nutrient mixture (1:1 ratio; DMEM-F12) and fetal bovine serum (FBS) were obtained from Gibco (Grand Island, NY, USA); the cell counting kit-8 (CCK8) was purchased from Dojindo Laboratories (Kumamoto, Japan); the LDH release assay was obtained from Beyotime Institute of Biotechnology (Shanghai, China); and the cell cycle assay caspase 3 activity assay kit and Annexin V/propidium iodide (PI) were purchased from Becton Dickinson Company (BD; Franklin Lakes, NJ, USA).

The polyclonal antibodies against GAPDH (2118), CHOP (2895S), PERK (3192s), P62 (5114S), P-ERK1/2 (54240), ERK1/2 (4695S), P-JNK1/2 (4668S), JNK1/2 (9252S), P-mTOR (5536S), mTOR (2983S) were acquired from Cell Signaling Technology (Boston, MA, USA); the polyclonal antibodies against BiP (ab21685), ATF6 (ab203119), p-IRE1 (ab48187) were purchased from Abcam (Cambridge, MA, USA). Vimentin (sc-6260) and N-cadherin (sc-7939) were obtained from Santa Cruz (Santa Cruz, CA). All other chemicals and reagents were analytical grade and were obtained commercially.

### Cell culture

TM4 cells, a mouse Sertoli cell line obtained from the ATCC, were cultured in DMEM/F-12 supplemented with 10% FBS and maintained at 37 °C in a humidified atmosphere with 5% CO_2_.

### Confocal immunohistochemistry

TM4 Sertoli cells were treated with ZEA for 24 h. The cells were fixed in 4% paraformaldehyde for 30 min at room temperature, permeabilized by using 0.5% Triton X-100 and then blocked with 5% BSA. The cells were incubated with phalloidin-tetramethy1 rhodamine isothiocyanate (TRITC) (1:50) and anti-LC3 antibodies. Finally, the slides were visualized under a confocal laser-scanning microscope (Leica TCS SP8; Leica, Germany).

### Western blotting analysis

The cells were harvested from the cultures, placed in the RIPA lysis buffer on ice (Beyotime, Haimen, China) and lysed by ultrasonication. The whole proteins were subjected to 10% sodium dodecyl sulfate polyacrylamide gel electrophoresis and then transferred to polyvinylidene fluoride membranes. The membranes were probed with the indicated primary antibodies at 4 °C overnight, washed and then incubated with secondary antibodies for 2 h at room temperature. The signal was developed by ECL detection system, and relative photographic density was quantitated by a gel documentation and analysis.

### Transmission electron microscopy (TEM)

The cells were fixed by using 2.5% glutaraldehyde at 4 °C for 24 h. Subsequently, the samples were washed with PBS (0.1 M, pH 7.4) and post-fixed for 20 min in 1% OsO4. The samples were dehydrated by using a series of ethanol solutions (20–100%) and embedded in an Epon: alcohol mixture (1:1) for 2 h. The samples were incubated overnight in a 37 °C oven. Thin sections (70 nm) were cut, placed on copper grids, and stained with a 2% uranyl acetate solution in a 1% solution of lead citrate for 30 min. A JEM 100CX transmission electron microscope, operated at 50–60 kV, was used to visualize the ultrastructure of the cells.

### Monodansylcadaverine (MDC) staining

MDC staining was used as a tracer to detect autophagic vesicles. Positive cells were colored in their perinuclear region, cellular autophagy was observed, and all acidic vacuoles were stained. Cell climbing slides were prepared overnight for group treatments, and 0.05 mM MDC (Shanghai Huzheng Industrial Co. Ltd.) was added to the sheets in a water bath at 37 °C for 15 min, washed three times with PBS, followed by immobilization with 4% paraformaldehyde for 15 min. Fluorescence microscope observation was then performed on an anti-fluorescence quenching slide to avoid the exposure to light.

### Transfecting mRFP-GFP adenoviral vectors

TM4 cells were plated in 24-well plates and transfected when they reached 50–70% confluence. mRFP-GFP-LC3 adenoviral vectors were purchased from HanBio Technology Co. Ltd. (HanBio, Shanghai, China). Adenoviral infection was performed according to the manufacturer’s instructions. The multiplicity of infection was 100. The cells were incubated with the adenovirus in DMEM/F-12 with no serum for 2 h at 37 °C. The transfected cells were incubated with DMEM/F-12 supplemented with fetal bovine serum overnight prior to ZEA treatment. Autophagy was observed under a confocal microscope (Leica TCS SP8 STED; Leica, Germany).

### Gene silencing with short interfering RNAs (siRNAs)

The ERK2 siRNA (6578S) was acquired from Cell Signaling Technology (Boston, MA, USA); Transfection of the siRNA oligonucleotides was performed using Lipofectamine® 2000 reagent (Invitrogen, Carlsbad, CA, USA). One day before transfection, the cells were plated in 500 μl of growth medium without antibiotics such that they will be 30–50% confluent at the time of transfection. The 20 pmol siRNA oligomer was diluted in 50 μl Opti-MEM without serum. Mixing the Lipofectamine 2000 gently before use, then diluted in Opti- MEM (1:50). The oligomer-Lipofectamine 2000 complexes was added to each well.

### Measurement of intracellular Ca^2+^

The level of intracellular Ca^2+^ was measured by using the fluorescent indicator Fluo-3 AM, according to the manufacturer’s protocol (Beyotime Institute of Biotechnology, Shanghai, China), with certain modifications. After exposure to ZEA for 24 h, the cells were collected, rinsed twice with PBS at room temperature, stained with Fura-3 AM dissolved in PBS for 30 min at 37 °C, and then detected by flow cytometry.

### Statistical analysis

The results are presented as the mean ± standard deviation (SD). Statistical data comparisons among groups were performed using a non-parametric, SPSS one-way analysis of variance, with p < 0.05 considered statistically significant. Each experiment was performed at least in triplicate.
