# Author Correction: Zearalenone altered the cytoskeletal structure via ER stress– autophagy– oxidative stress pathway in mouse TM4 Sertoli cells

**DOI:** 10.1038/s41598-020-67552-y

**Published:** 2020-06-25

**Authors:** Wanglong Zheng, Bingjie Wang, Mengxue Si, Hui Zou, Ruilong Song, Jianhong Gu, Yan Yuan, Xuezhong Liu, Guoqiang Zhu, Jianfa Bai, Jianchun Bian, ZongPing Liu

**Affiliations:** 1 College of Veterinary Medicine, Yangzhou University, Yangzhou, 225009 Jiangsu China; 2 Jiangsu Co-innovation Center for Prevention and Control of Important Animal Infectious Diseases and Zoonoses, Yangzhou, 225009 Jiangsu China; 3 Joint International Research Laboratory of Agriculture and Agri-Product Safety, the Ministry of Education of China, Yangzhou University, Yangzhou, 225009 China; 4 Kansas State Veterinary Diagnostic Laboratory, Kansas State University, 1800 Denison Avenue, Manhattan, KS 66506 USA

Correction to: *Scientific Reports* 10.1038/s41598-018-21567-8, published online 20 February 2018

This Article contains errors.

As a result of errors in figure assembly, Figure 2C the merged image for ZEA 10 µM is a duplication of the merged image for ZEA 1 µm. The correct Figure 2C is included below as Figure 1.

**Figure 1:**
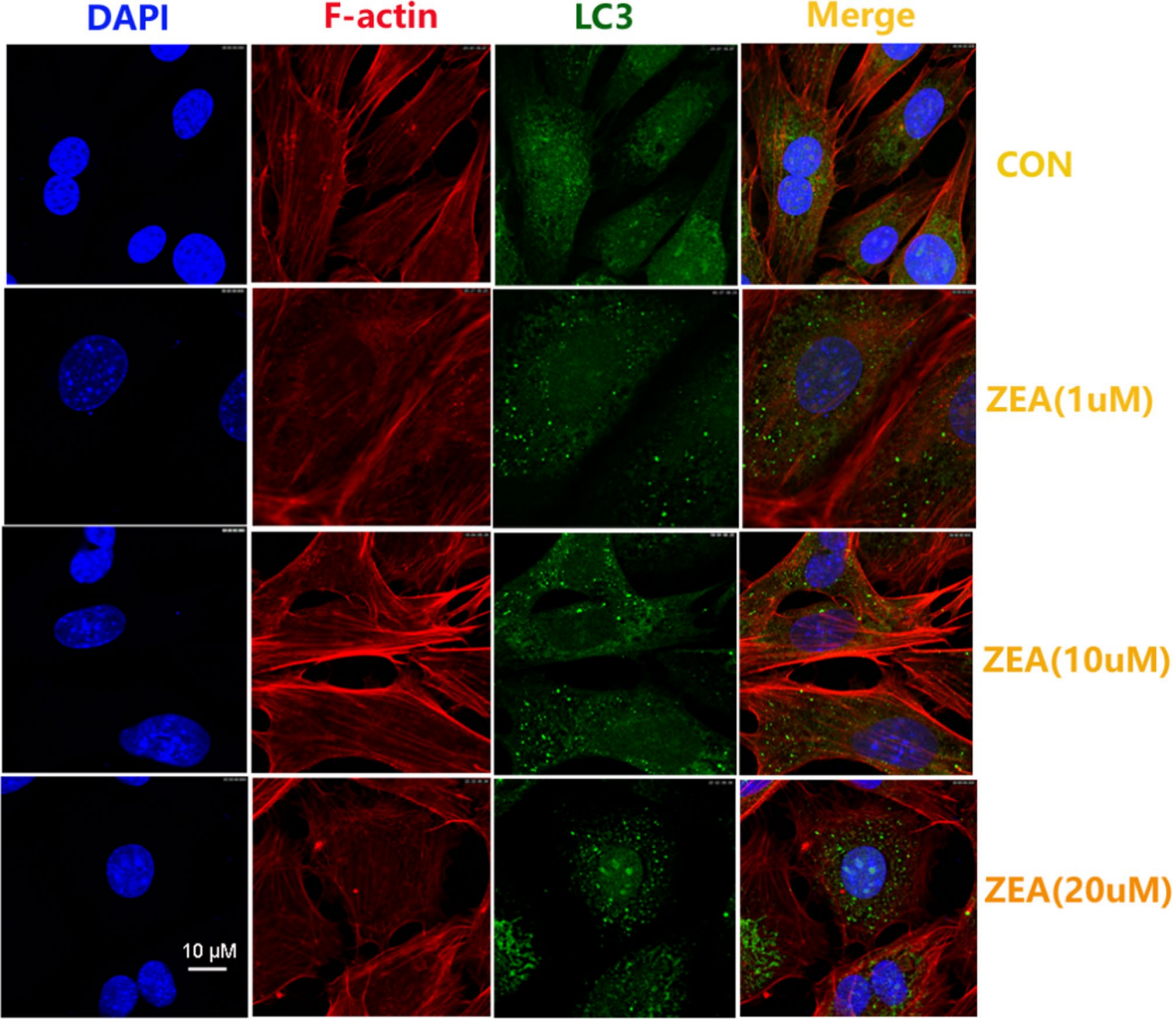
.

Furthermore, in Figure 4, all images for RAP 5 µM are a duplication of ZEA 10 µm images from Figure 1C. The correct Figure 4 is included below as Figure 2.

**Figure 2:**
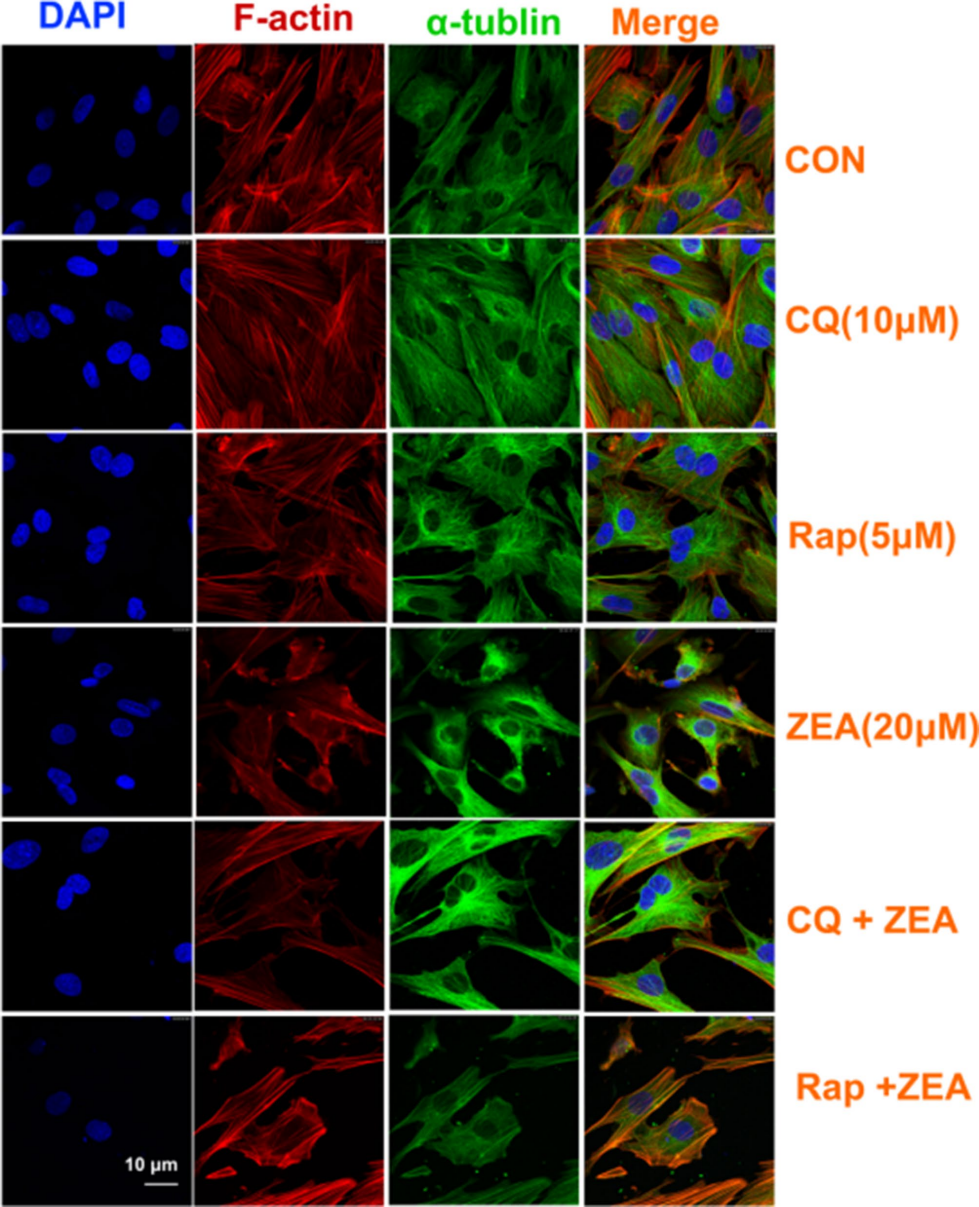
.

Finally, in Figure 5E, all images for CON are a duplication of CQ images from Figure 4, and all NAC images are a duplication of CON from Figure 1C. The correct Figure 5E is included below as Figure 3.

**Figure 3:**
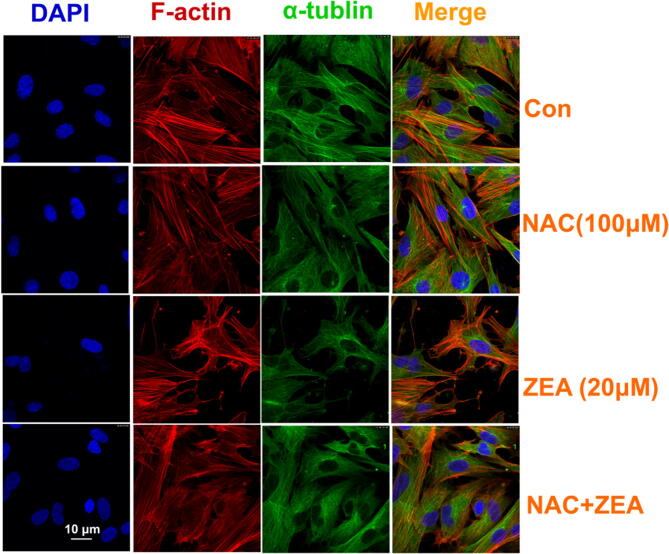
.

The overall conclusions of the Article are unaffected by these changes.

